# Retraction: Begum *et al.* Potential Impact of Multi-Walled Carbon Nanotubes Exposure to the Seedling Stage of Selected Plant Species. *Nanomaterials* 2014, *4*, 203–221

**DOI:** 10.3390/nano5010268

**Published:** 2015-03-02

**Authors:** Nanomaterials Editorial Office

**Affiliations:** MDPI AG, Klybeckstrasse 64, Basel CH-4057, Switzerland; E-Mail: nanomaterials@mdpi.com

We have become aware that a substantial part of the main text of [1] is copied from multiple other publications. In total, 46% of the main text was taken from publications by the same authors [2,3] and 10% from other papers [4,5]. Because of the extent of text taken verbatim from previously published articles, we have made the decision to retract the article. All the authors of [1] have agreed to this decision. This paper is thus declared retracted and shall be marked accordingly for the scientific record.

MDPI is a member of the Committee on Publication Ethics (COPE) and takes the responsibility to enforce strict ethical policies and standards very seriously. We aim to ensure the publication only of truly original scientific works. MDPI would like to apologize to the readers of *Nanomaterials* that this case remained undetected until now. We sincerely appreciate the efforts of anyone who brings matters of plagiarism to our attention in an effort to maintain scientific integrity.
